# Temperature and evaporative water loss of leaf-sitting frogs: the role of reflection spectra

**DOI:** 10.1242/bio.021113

**Published:** 2016-10-28

**Authors:** Francisco Herrerías-Azcué, Chris Blount, Mark Dickinson

**Affiliations:** Photon Science Institute, School of Physics and Astronomy, The University of Manchester, Manchester M139PL, UK

**Keywords:** Frogs, Thermoregulation, Reflection, Infrared, Modelling, Spectrum

## Abstract

The near infrared reflection peak in some frogs has been speculated to be either for enhancing crypticity, or to help them with thermoregulation. The theoretical background for the thermoregulatory processes has been established before, but little consideration has been given to the contribution from the frogs' reflection spectra differences. In this investigation, the reflection spectra from a range of different species of frogs were taken and combined with precise surface area measurements of frogs and an approximation to the mass transfer coefficient of agar frog models. These were then used to simulate the temperature and water evaporation in anurans with and without the near infrared reflective peak. We have shown that the presence of the near infrared reflection peak can contribute significantly to the temperature and evaporative water loss of a frog. The significance of the steady-state temperature differences between frogs with and without the near infrared reflection peak is discussed in a realistic and an extreme scenario. Temperature differences of up to 3.2°C were found, and the rehydration period was increased by up to 16.7%, although this does not reduce the number of rehydration events between dawn and dusk.

## Abstract (precis)

**Summary:** We show that the presence of a reflection peak in the NIR in some frogs can contribute to a temperature difference of up to 3.2°C, which extends the period they can go without rehydration by over an hour (22.7%).

## INTRODUCTION

Since the discovery of pterorhodin in the skin of *Agalychnis dachnicolor* (formerly *Pachymedusa dachnicolor*) (Emerson et al., 1990), which replaces melanin and produces a characteristic peak in the near infrared (NIR) part of the reflection spectrum, the relevance of NIR-enhanced reflection on different frog species has been an open question.

The leaves these frogs often sit on also show a reflective peak in the near infrared, similar to that from frogs containing pterorhodin, which would suggest that crypticity plays an important role (Schwalm et al., 1977). However, there are no reports of leaf-sitting frog predators that can see in that part of the spectrum, which makes IR crypticity seemingly unimportant.

A reduction in the amount of light absorbed by an animal will decrease the amount of energy gained, and therefore there could be a link to thermoregulation. Emerson et al. (1990) briefly mention an estimated 2°C temperature difference between frogs with and without NIR-enhanced reflectance, but they only use a very rough calculation and only take a single absorptance difference. Here we report on a detailed program to simulate the thermoregulatory processes in frogs, taking into account the reflection spectra differences, to analyse whether there is in fact a temperature difference, and how it could reflect on the water balance of the frog.

## SIMULATION RESULTS

### Fully exposed, horizontal frog

**Fig. 1.**
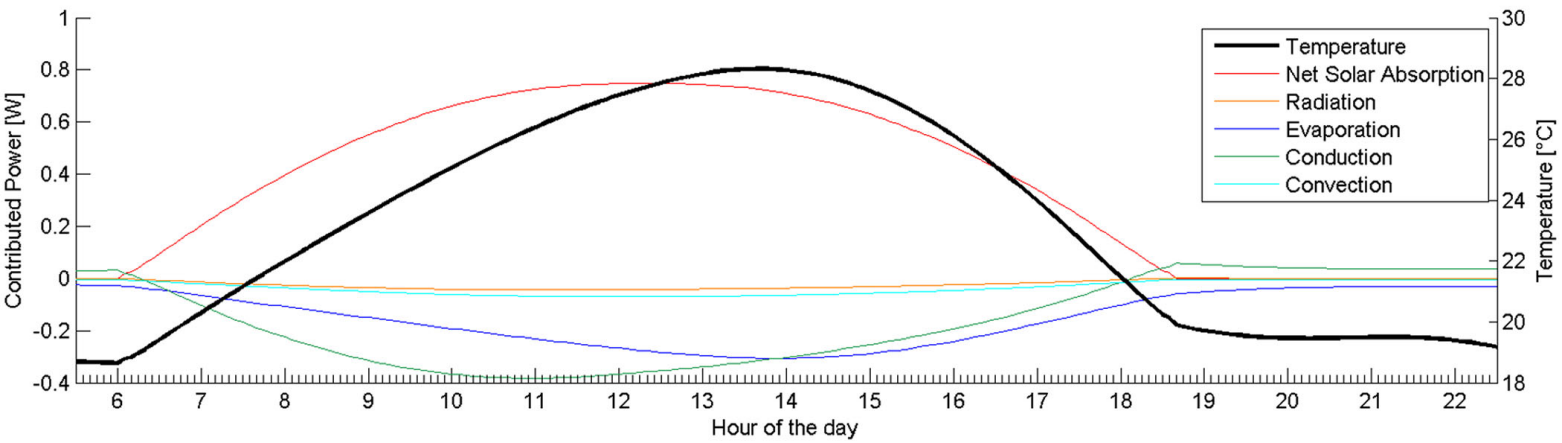
**Sample simulation result in fully exposed scenario.** Power contributions (left axis), and the resulting steady-state frog temperature (right axis) plotted against time. The plot corresponds to *C. craspedopus* (with pterorhodin) sitting flat and with no cover.

An example of the output generated for a fully exposed, horizontal frog is shown in Fig. 1. Note the scale of the temperature, as well as the shape of the temperature profile.

In this scenario, the *C. craspedopus* reached a maximum temperature of 28.3°C, the black frog reached a maximum of 30.5°C and the black IR-reflecting frog, reached 27.7°C (the latter two are taken from separate simulations not shown in Fig. 1). When run for different frog species, the difference between the highest and lowest maximum temperatures was 1.7°C.

**Fig. 2.**
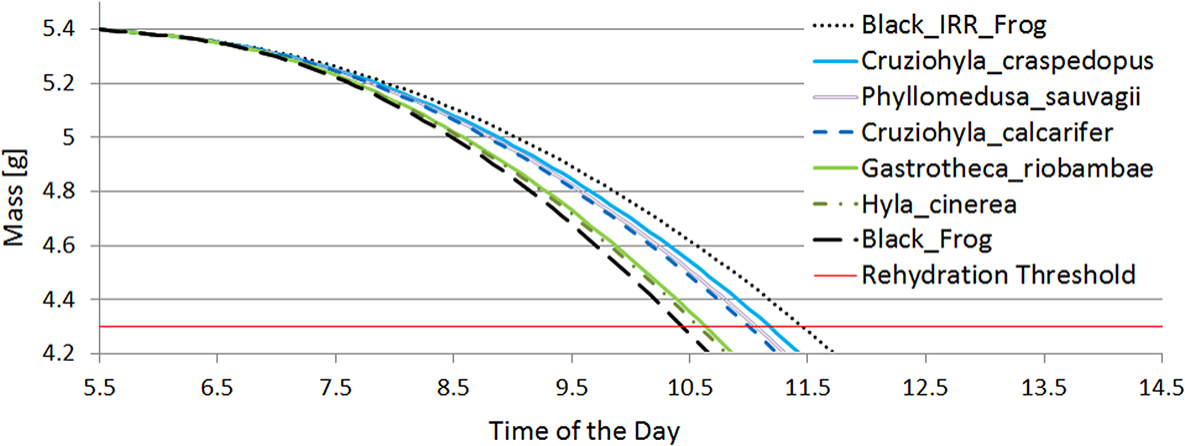
**Mass of the frogs through the simulated period in the fully exposed scenario.** It is assumed that water evaporation is the only source of change. Rehydration threshold was set at 4.3 g.

The mass of the frog versus time of day is shown in Fig. 2, where it can be seen that a completely black frog would need to rehydrate after about 4.3 h of sun exposure, whereas the black IR-reflecting frog would need rehydration after 5.4 h. The difference between the maximum exposure times before rehydration in real spectra simulations was 0.6 h.

### Partially occluded frog

**Fig. 3.**
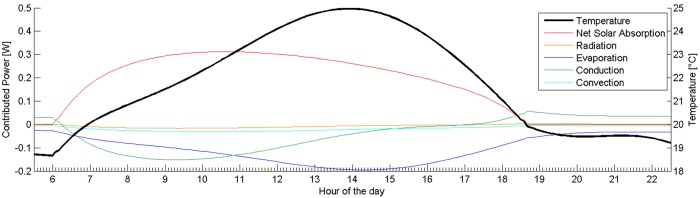
**Sample simulation result in partially occluded scenario.** Power contributions (left axis), and the resulting steady-state frog temperature (right axis) plotted against time. The plot corresponds to *C. calcarifer* (with pterorhodin) sitting on a leaf with a 65% shade, zenith angle of 70°, and azimuth angle of 20°. Note the asymmetry of the solar radiation contribution.

An example of the output generated for a partially occluded frog is shown in Fig. 3. Note the scale of the temperature, as well as the shape of the temperature profile and how this differs to that in Fig. 1. The shape is mainly due to the projected area of the frog changing throughout the day.

**Fig. 4.**
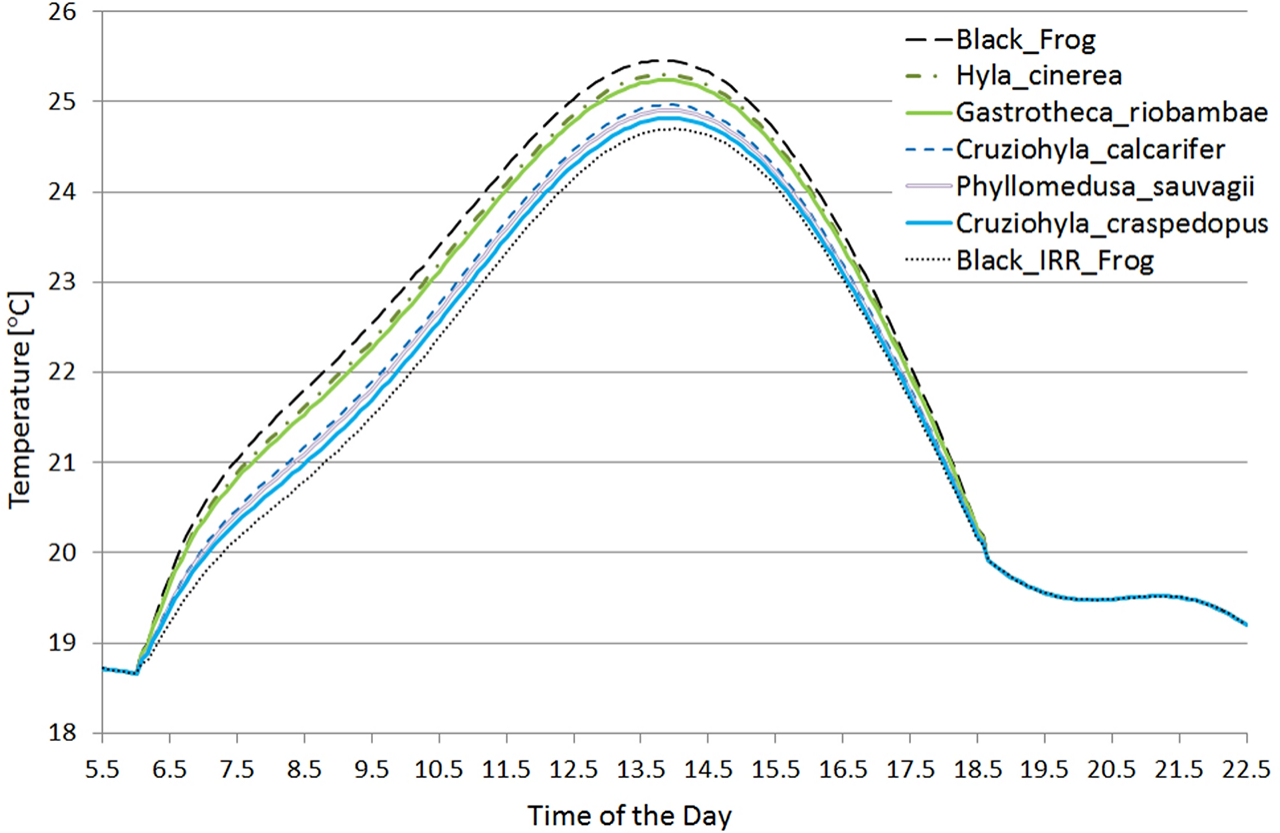
**Temperature of the frogs through the simulated period in the partially occluded scenario.** Note how the black and black IR-reflecting frogs serve as an envelope for the other spectra (the *Hyla* and *Gastrotheca* species do not present reflectivity in the near infrared).

In this scenario the maximum temperature of the *Cruziohyla calcarifer* frog was 25.0°C, for the black frog 25.5°C, and for the black IR-reflecting frog 24.7°C. When run for different frog species, the difference between the highest and lowest maximum temperatures was 0.5°C. Fig. 4 shows the temperature profile for all seven frogs used in the simulation.

The shape of the temperature profile for all the frogs modelled are clearly very similar, and at dawn and dusk they can all be seen to converge to the same point, as no solar radiation is being absorbed.

In this scenario, a completely black frog would need to rehydrate after about 5.9 h, and the black IR-reflecting frog would need rehydration after 6.8 h. In the case of the real frogs, the time difference between the maximum exposure times before rehydration is necessary was 0.6 h.

## DISCUSSION

As can be seen in Fig. 4, the temperature of the completely absorbing frog (black) and the one built to absorb all but the near infrared light (black IR-reflecting) serve as an envelope to the temperatures of the real frogs. This indicates that it is safe to assume that these spectra serve as the limiting values.

For the completely exposed scenario, the differences in temperature (at most 2.8°C) and in maximum exposure time before rehydration (at most 22.7%) suggest that the changes in the spectrum can contribute significantly to the thermoregulation process.

Interestingly, although the temperature difference between frogs with the IR reflective peak and without it reduces drastically in the partially occluded scenario, the difference in the time that they can be exposed before rehydration is necessary does not diminish as much. The difference in temperature between the black and black IR-reflecting reference frogs only accounts for a maximum of 0.8°C. On the other hand, this is reflected as a 14.2% prolongation of the time they can be exposed, which still suggests a significant increase in their water holding efficiency.

In the real frogs, these reduce to a maximum 0.5°C temperature difference, and an 9.3% prolongation of the time they can be exposed, which could still be a relevant factor to avoid movement during the day.

However, the difference in rehydration time is not enough to change the number of times the frog would need to rehydrate between dawn and dusk, which again makes the role of IR reflectance in thermoregulation unclear. There are still other possibilities that could explain the purpose of IR reflectance, and these need to be explored further before any single evolutionary trigger can be selected.

## CONCLUSIONS

Results are presented for two scenarios (fully exposed and partially occluded) and suggest that the infrared reflection spectrum does generate changes in the thermoregulatory processes, and these discrete changes can directly affect the water balance of the frogs. Although the resulting temperature difference appears to be negligible (at most 0.8°C in a realistic scenario), it is accompanied by a larger difference between rehydration periods (up to 14.2% in the same circumstances) which, although significant, does not reduce the number of rehydration events needed between dawn and dusk.

Whether the effect on steady-state temperature or water conservation are enough to trigger the evolution of infrared reflectivity or not remains unclear and further studies on its role in crypticity have to be made before a final conclusion is reached.

An accurate and complete simulator for predicting temperature and water loss for anurans with different reflection spectra has been developed and tested on a range of species with and without the near infrared reflection peak.

## MATERIALS AND METHODS

### Animal handling

Animal handling was performed by the trained staff of the Vivarium at The Manchester Museum, and conformed to all regulations and animal welfare laws.

### Thermoregulation model

The theory behind thermoregulation has not developed significantly since evaporative water loss (EWL) was introduced by Richard Tracy (Tracy, 1976, 1982) onto the model presented by Porter and Gates (1969). It has since been considered that the main factors affecting the steady-state temperature of a frog are absorbed radiation, emitted radiation, conduction, convection and evaporative water loss. Since frogs are ectotherms, their metabolism does not contribute significantly to the power balance, and it is usually considered to even out with the power lost through respiratory water evaporation (Tracy, 1976). For this reason it is neglected in this study.

Absorbed radiation is the main heat input, and since this is directly dependent on the specific absorption spectra of the frog being modelled, it should be standard practice to include absorption spectra in any thermoregulation model. However, reflection spectra differences have not been directly taken into account in the literature. Since we aim to clarify the purpose of infrared reflectance, including the absorption and reflection spectra becomes even more relevant.

It is important to note that neo-tropical tree frogs sleep during the day on or under leaves, from which no water intake is available, and therefore we assume no water intake by the frog during the simulation period.

#### Absorbed radiation

The main contribution to absorbed radiation is usually direct solar radiation, but also includes diffuse and reflected light. The amount of radiation absorbed by the frog depends greatly on its dorsal area, how exposed it is, and on the overlap of its absorption spectra and the emission spectra of the sun. This is where the differences in reflection spectra between frogs need to be taken into account. It is assumed that all radiation not being reflected is absorbed, i.e. that there are no other processes occurring, and that the penetration depth is small enough that transmission can be neglected.

The solar spectrum was simulated using a simplified version of Gueymard's ‘Simple Model of the Atmospheric Radiative Transfer of Sunshine’ (SMARTS) (Gueymard, 1995), and it takes the global position into account by using the PSA algorithm (Blanco-Muriel et al., 2001). Both codes were translated into MATLAB (The MathWorks Inc.) to run natively and work swiftly with the rest of the simulator. This results in the generation of a vector that contains the solar irradiance spectrum for the direct radiation (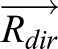), and another for the diffuse and reflected radiation (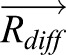), which can then be used to calculate the power absorbed (*P_sol_*) as
(1)
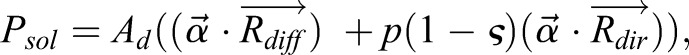


where 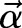 is the frog's absorption spectrum, *A_d_* is its dorsal area, *p* is a projection factor that changes with the solar position, and *ς* is a shade factor that goes from 0 (completely exposed) to 1 (fully shaded).

##### Emitted radiation

The simulation assumes that the frog radiates energy as
(2)
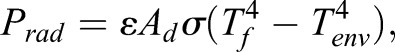
where *P_rad_* is the power radiated by the frog, *ε* is its emissivity (in anurans usually reported around 0.96; Tracy, 1982), *σ* is the Stephan–Boltzmann constant, *T_f_* is the temperature of the frog, and *T_env_* is the temperature of its surrounding environment, both in K.

##### Conduction

For big animals, the time that heat takes to be conducted from the core to the integument and vice versa has to be taken into account (Tracy, 1976; Porter and Gates, 1969) because it adds a thermal inertia to the system. In small animals, however, the core and outer shell can be treated as one because their small mass provides negligible inertia (Christian et al., 2006). Conduction to the substrate takes place only through the ventral area (*A_v_*) of the frog, and the heat transfer is simulated as a flat slab of cross sectional area equal to *A_v_*. As such, the power that the frog gains through conduction (*P_cond_*) is described by
(3)
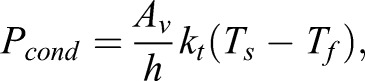
where *h* is the thickness of the frog, *k_t_* is the thermal conductivity (this can be of the frog or of the substrate; in simulations, the lowest value is taken), and *T_s_* is the temperature of the substrate on which the frog is sitting.

##### Convection

The heat that the air surrounding the frog takes away is simulated as
(4)
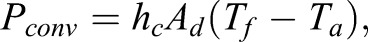
where *P_conv_* is the power the frog loses through convection, *h_c_* is a heat transfer coefficient, which has been approximated in the past as a function of wind speed (Porter and Gates, 1969; Mitchell, 1976), and *T_a_* is the temperature of the air surrounding the frog.

#### Evaporative water loss (EWL)

Water evaporation through the skin is one of the main elements controlling the cooling process in most anurans (Bartelt and Peterson, 2005). Tree frogs usually sit in a water-conserving posture that creates a water-tight seal around their ventral area, limiting the evaporation surface to their dorsal area. The rate at which water is being evaporated will vary greatly between species due to skin properties (Tracy et al., 2010), and it will also change depending on the water vapour pressure difference between the frog and the air around it, and of course also on wind velocities (Tracy and Christian, 2005). The evaporation rate for most anurans can be simulated using a free water surface (Tracy, 1976) and as such, the frog's water vapour density (*ρ_s_*) can be directly imputed from the saturation value, which can be found in tables (http://www.engineeringtoolbox.com/water-vapor-saturation-pressure-air-d_689.html). The surrounding air will have a water vapour density (*ρ_a_*) equal to the product of the saturation value and the relative humidity (*RH*).

The power exchanged through EWL is given by
(5)
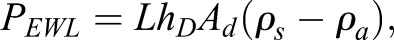
where *L* is the latent heat of water (L=2260 kJ/kg), and *h_D_* is the mass transfer coefficient, which accounts for the variation in evaporation rates due to species, shape and wind speed.

The mass transfer coefficient was measured in agar models following the experimental method described by Tracy (1976). Five differently shaped models were measured at five different wind velocities and two orientations relative to the wind direction. Two of the models were based on *Cruziohyla calcarifer* frogs of snout-vent lengths of 6.1 cm and 4.3 cm, two were based on *Cruziohyla craspedopus* frogs of 4.8 cm and 3.1 cm, and the last model was a hemisphere of 3.1 cm diameter.

A total of seven parameters were measured: mass, time, dorsal area, wind speed, temperature of the frog, air temperature and relative humidity. For each experiment the wind tunnel was set at a speed and left to stabilize. Then, a model or group of models were introduced to the wind tunnel and set at an angle of either 0° or 90° relative to the wind direction. Once the temperature change stabilized, the model's mass and temperature, as well as the air's temperature and relative humidity, were monitored at regular intervals for a set period of time. The frog models had to be momentarily removed from the wind tunnel to be weighed each time.

The experiments took place in the facilities of the School of Mechanical, Aerospace and Civil Engineering at the University of Manchester, where the ‘Armfield Tunnel’ was chosen for its capacity to produce steady low wind speeds (1 m/s to 5 m/s) needed for this experiment. The speed was measured with a precision of 0.1 m/s. The frog models were weighed using a Kern EMB500-1 precision scale with an accuracy of 0.1 g. The surface area of the models was the same as the moulds printed for them, for which accuracy similar to that of the 3D rendering technique is expected, i.e. an error of 2.5%. The temperature of the frog was measured with an 8889 IR thermometer with an accuracy of 0.5°C. The air temperature and relative humidity were monitored with an HTD-625 hygrometer-thermometer with an accuracy of 0.5°C and 2% respectively. Measurements were taken every 5 min for wind speeds around 5 m/s, every 7.5 min for velocities around 3 and 4 m/s, and every 10 min for speeds around 1 and 2 m/s. The orientation of the models was only measured qualitatively.

A total of 80 complete measurements were acquired at 1.46 m/s, 2.12 m/s, 2.82 m/s, 3.8 m/s and 4.63 m/s. All five differently shaped models were tested at a 0° orientation (with the head pointing towards the wind). At 90° (perpendicular to the wind), only the models of the two *C. calcarifer* and the bigger *C. craspedopus* were tested.

It is difficult to maintain wind speeds lower than the m/s level in wind tunnels, and therefore low velocity measurements are very difficult to obtain. Since in tropical forests wind speeds are usually of the order cm/s, being able to extrapolate values of the mass transfer coefficient is necessary.

It is important to note that *h_D_* should have a positive and non-zero intercept, which represents the water being evaporated from the frog when there is no wind. Also, since with no wind the evaporation rate would not depend on shape but only on dorsal area, the data for all models should converge to a single intercept. On the other side, as wind velocities increase the mass transfer coefficients should diverge between different shapes, as Tracy (1976) suggests.

Cussler (2009) proposes a relationship between the mass transfer coefficient and the wind velocity of the form *h_d_*∼*v^1/2^*, but when applied to our data negative intercepts were found. A linear or a quadratic relationship result in positive intercepts, but with a very wide spread.

**Fig. 5.**
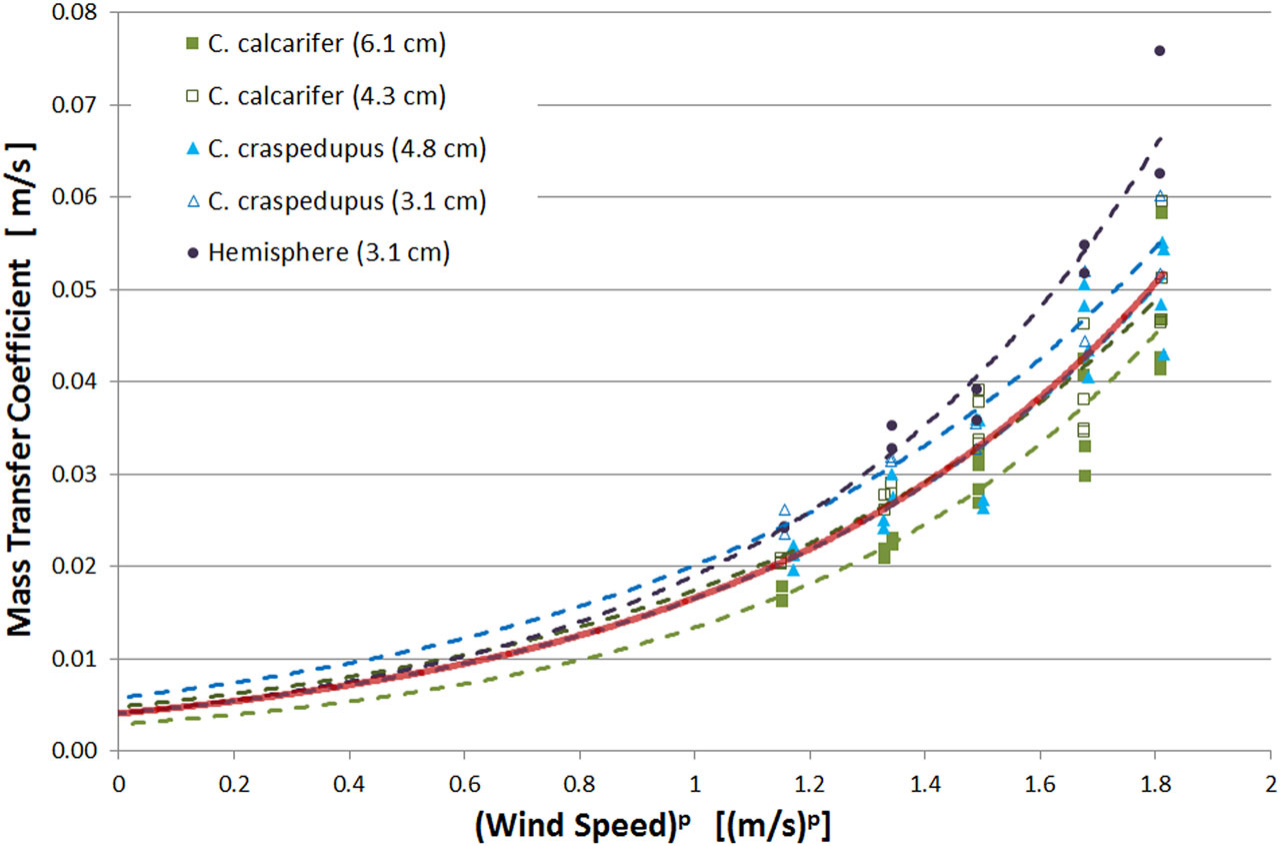
**Mass transfer coefficient plotted against the wind speed to the power of 0.39.** Dashed lines are fitted curves for single models, and the thick red line includes all the data. All fitted lines are of the form described in Eqn 6. Note that the intercept points converge and diverges for different shapes at higher wind speeds.

To emphasize the low wind speed interpolation and taking into account the divergence between different shapes at higher wind velocities, an experimental fit is proposed (see Fig. 5), which follows a relationship of the form
(6)
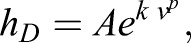
where *v* is the wind speed in m/s and *A*, *k* and *p* are experimentally determined constants.

A value of *P*=0.39 was found by maximizing *R^2^* in a fit for all models, and the other two parameters were allowed to change between frog models, reaching an average of *A*=0.0041 m/s and *k*=1.40 (s/m)*^p^*. It is imperative to stress however, that this relationship is only proposed to fit the data with an emphasis on the intercept point, which is essential in the simulation, but there is no theoretical basis for it.

#### Surface areas

All the thermoregulatory processes are affected by the surface area of the frogs, whether it is ventral area or dorsal area. During the simulation these areas can be approximated in different ways. One method is to model the frog as a hemisphere of a given radius, estimated from the mass of the frog, or inferred from the snout-vent length. Alternatively, actual surface areas related to a specific frog species can be used if these are known.

To address this issue, a non-invasive technique to measure the surface area of the frogs was developed by rendering a 3D model of the specimen from a series of photographs taken at different angles. The technique was tested in the two different species of the *Criziohyla* genus, and was found to be accurate to 1.2% in snout-vent measurements, and 2.5% in surface and volume measurements.

In collaboration with the Vivarium at the Manchester Museum, a total of 23 *C. craspeopus* and 20 *C. calcarifer* of different sizes and at different stages of development were photographed and their 3D models rendered in order to measure their surface areas and volume.

The *C. craspedopus* specimens ranged from 3.1 cm snout-vent and 2 g, to 5.8 cm snout-vent and 15 g. This covers sizes from frogs a few weeks after hatching to nearly fully grown adults. The *C. calcarifer* specimens ranged from 3.3 cm snout-vent and 3 g, to 6.4 cm snout-vent and 23 g, which also covers from very young froglets up to fully developed male adults, which are usually a bit smaller than adult females.

The specimens were placed on a turn table and slowly rotated while photographs were taken at no more than 15° intervals and from two different heights (∼20 and 40 cm). Animal handling was performed by the trained staff of the Vivarium at The Manchester Museum, conform to all regulations and animal welfare laws.

The 3D models were rendered using Smart3DCapture 3.1.1 (https://community.acute3d.com/smart3dcapture-free-edition), which accepts a series of photographs from different angles as input, reconstructs the photographed object and outputs a mesh. The mesh was further cleaned and corrected using Blender 2.73 (http://www.blender.org). The finalized mesh is a series of triangles with vertices spatially localized, and so it is easy to calculate areas of very complex shapes. The surface and volume of the mesh was computed using NeuroMorph toolkit (Jorstad et al., 2014).

The accuracy of the technique was evaluated either by comparing the rendered size of objects with regular shapes, known density or with printed 3D models, in which case the mesh size of the pre-print and the post-print version were compared. In all cases, the absolute average error was under 1.2% for linear measurements (i.e. snout-vent) and 2.5% for surface and volume measurements. See Herrerías-Azcué (2015) for further detail.

##### Mass inference

Interpolation of surface area from mass has already been reported in the literature (Tracy, 1976; Hutchison et al., 1968; McClanahan and Baldwin, 1969), often by fitting the surface area to a power function of the mass (*w*):
(7)
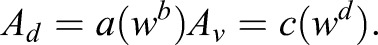


**Fig. 6.**
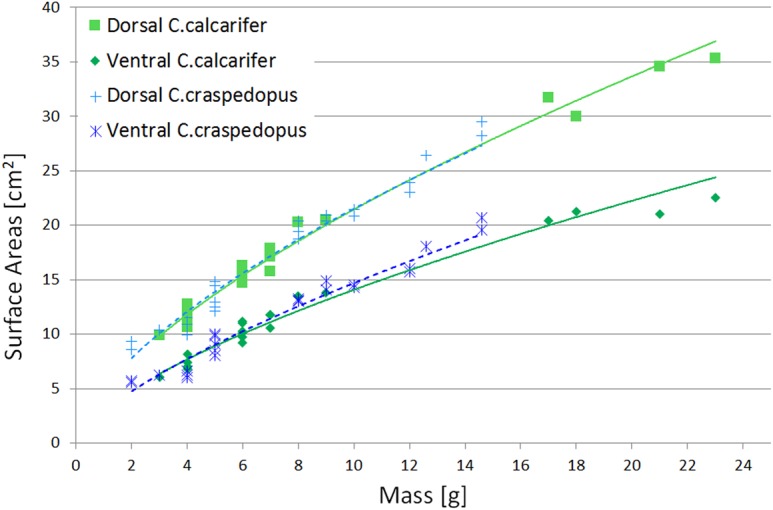
**Surface areas plotted against the mass of the frog.** Fitted lines follow the power function described in Eqn 7. Note how closely overlapped the fitted lines are, which suggests very similar surface area in both species.

The constants *a*, *b*, *c* and *d* are determined experimentally by fitting a curve to the experimental data. Fig. 6 shows the acquired measurements of the two species with masses ranging from 2 g to 23 g and the power fit corresponding to each of them. For *C. calcarifer*, the coefficients were found to be *a*=4.80, *b*=0.65, *c*=3.08 and *d*=0.66. For *C. craspedopus*, the coefficients were found to be *a*=5.04, *b*=0.63, *c*=2.92 and *d*=0.70.

##### Snout-vent inference

It is natural to expect a relationship between the snout-vent length of a frog, a measure of its size, and its surface area. Furthermore, it is a measurement that is usually gathered in any observation of a species, so it is a very convenient datum from which to start.

**Fig. 7.**
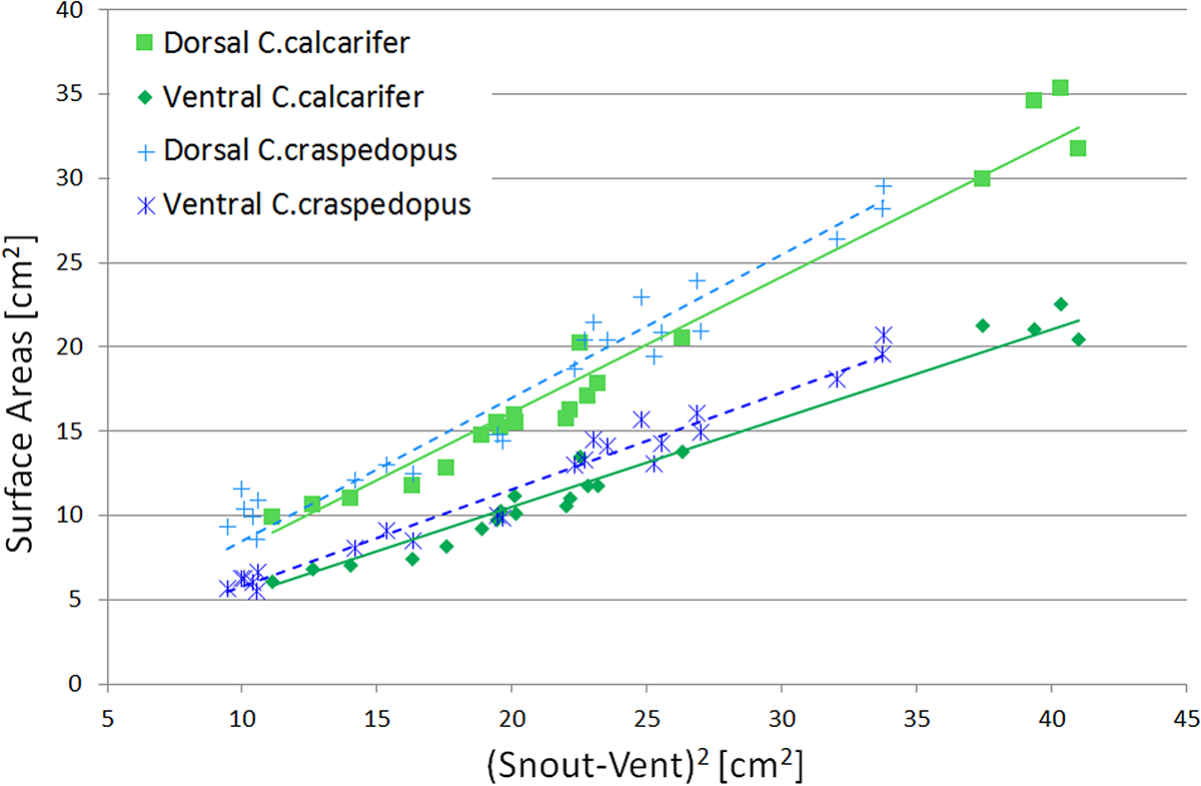
**Surface areas plotted against the snout-vent length squared.** Fitted lines follow the function described in Eqn 8. Note how the lines are clearly separated, which suggests clearly distinct surface area between species.

As can be seen in Fig. 7, there is a clear link between the snout-vent length squared and the surface areas, for which the interpolation proposed is simply
(8)
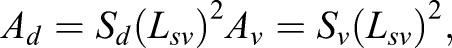
where *L_sv_* is the snout-vent length, and the constants *S_d_* and *S_v_* are determined with the linear fit. The coefficients found for *C. calcarifer* were *S_d_*=0.81 and *S_v_*=0.53 whereas for *C. craspedopus*, they were found to be *S_d_*=0.85 and *S_v_*=0.58.

#### Simulation scenarios

**Fig. 8.**
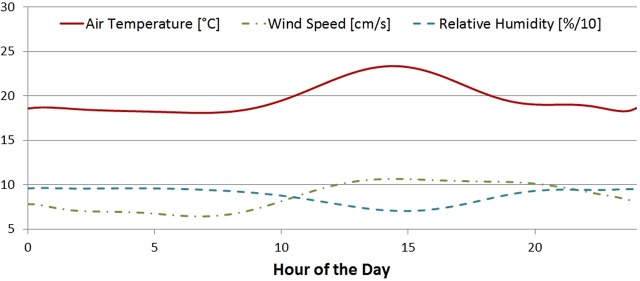
**Ambient profile used in the simulations.** Ambient temperature, wind speed and relative humidity throughout the simulated day. The relative humidity was divided by a factor of 10 so that it could be shown next to the wind speed.

The simulator receives inputs that will determine the heat transfer mechanisms already described, and finds the equilibrium temperature of the frog at set intervals for the whole simulation period. Since the air temperature, wind speed and humidity are very likely to change in a long simulation, an ambient profile can be introduced to yield more accurate results. The ambient profile used for the simulations reported here is shown in Fig. 8, and was obtained from interpolated meteorological reports from Amazonian forests (Lowman and Rinker, 2004), combined with the wind speed differences between substrate level and at the canopies described in (Baynton et al., 1965).

Two different scenarios were simulated. One of them proposes the most extreme circumstances for a frog, where it is fully exposed to the sun and sitting flat on the ground. The other one is perhaps a more realistic scenario, and it represents a partially occluded frog. The shade factor was set to *ς*=0.65, it was perched on a leaf at an angle of 70° from the vertical leaning to the NNE. All other inputs were the same in both scenarios, which means that the only difference between the frogs simulated is their reflection spectra.

**Fig. 9.**
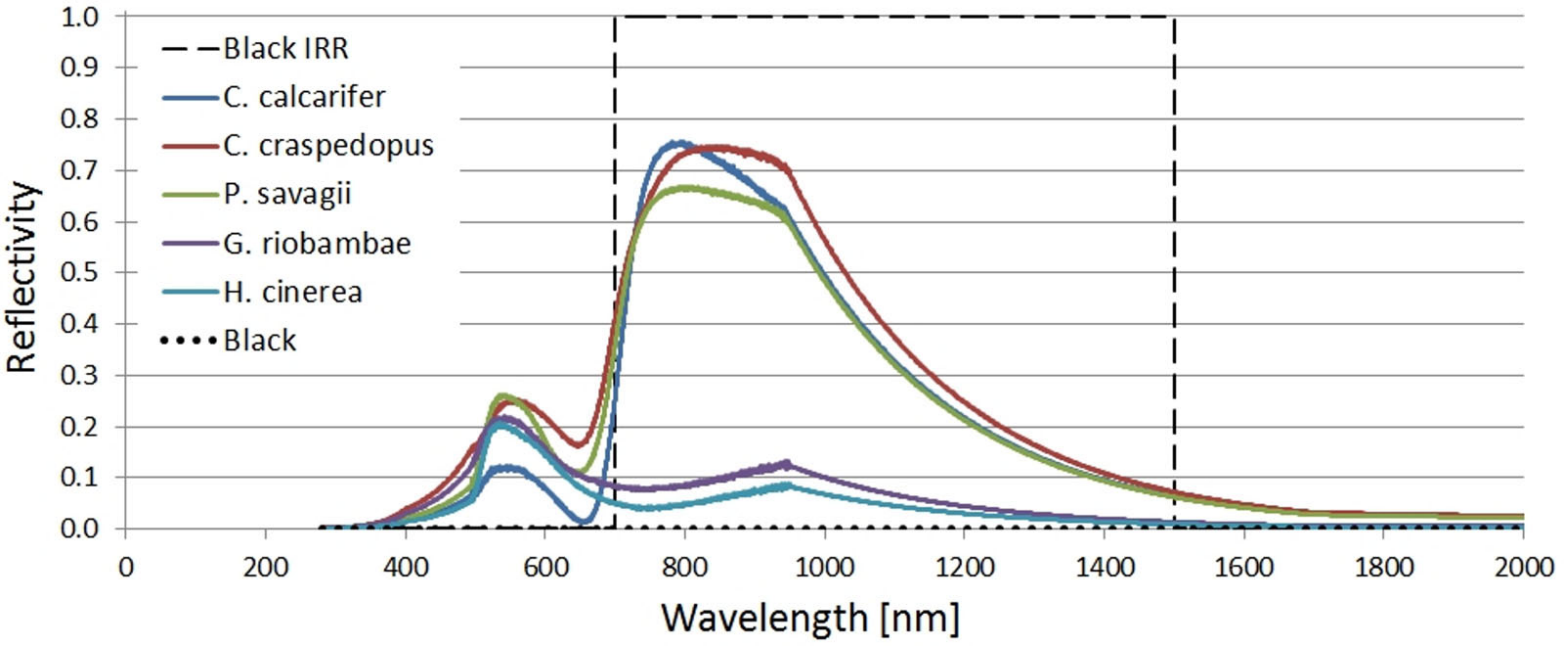
**Examples of reflection spectra of frogs.** Note how some of the used spectra show the near infra-red reflection peak at ∼700 nm and some do not. The dotted and dashed lines are extreme reference spectra built for a completely black frog, and a black infrared reflective frog. An exponential fall of was forced after 1000 nm.

The sample spectra shown in Fig. 9 were measured from specimens being held at The Manchester Museum's Vivarium using an Ocean Optics USB4000-VIS-NIR spectrometer. Due to the lack of accuracy of the spectrometer outside of the 450-950 nm range, the measurements were truncated at 450 nm and 950 nm, and an exponential fall off (*e*^−5^) was added at the edges. Most of the radiated energy of the sun that is available for absorption by the frog falls within this range, and spectra of different species has been found to be very similar out of it (Emerson et al., 1990), for which changes in temperature between species due to absorption out of that range would be even less relevant.

The depicted spectra are the result of the average of five measurements, smoothed with a 10 wide boxcar method. Water absorption bands are not visible due to the truncation and exponential fall-off.

Three species with the characteristic IR reflectance peak (*C. calcarifer*, *C. craspedopus* and *Phyllomedusa sauvagii*) and two without it (*Gastrotheca riobambae* and *Hyla cinerea*) were considered, as well as a completely absorbent (black) spectrum (*α*=1) and a black IRR-reflective spectrum (*α*=0 if 700 nm≤*λ*≤1500 mm and *α*=1 in any other case) used as references.

The program internally converts these reflection spectra (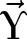) to absorption spectra (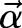) using
(9)
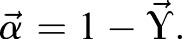


The frogs' size, global position and thermal properties were fixed for all spectra.

The significance of the temperature differences in the frogs was evaluated also through its effect on water conservation, as previously suggested in Tracy et al. (2010). As the simulation progresses the mass of the frog decreases due to EWL. Since they can hold up to 20% of their weight in their bladder (Shoemaker et al., 1989), a ‘rehydration boundary’ was set at 80% of their weight, and the time elapsed before this limit is reached is quoted. After that, the frog would necessarily need to rehydrate, which implies moving and being exposed while water is being absorbed. We refer to this as a rehydration event.
